# What’s going on with teleworking? a scoping review of its effects on well-being

**DOI:** 10.1371/journal.pone.0305567

**Published:** 2024-08-19

**Authors:** Mattia Vacchiano, Guillaume Fernandez, Rita Schmutz

**Affiliations:** 1 Department of Sociology, University of Geneva, Ginevra, Switzerland; 2 Swiss Centre of Expertise in Life Course Research LIVES, Lausanne, Switzerland; 3 Institute of Social Sciences, University of Lausanne, Lausanne, Switzerland; Instituto Federal Goiano, BRAZIL

## Abstract

Studies of teleworking and well-being increased dramatically during the COVID-19 pandemic. This article aims to provide an overview of this emerging body of knowledge. Following the PRISMA guidelines, we performed a scoping review using Social Sciences Citation Index (Web of Science), Sociological Abstracts (PROQUEST), and SocINDEX with full text (EBSCOhost). Articles published in English up to December 2022 were included. The result was a total of 2695 potentially relevant studies. After a double-screening procedure, 132 studies were chosen for data extraction. A content analysis was carried out to provide a summary of the social mechanisms linking teleworking to indicators of well-being related to mental health and quality of life. A complex picture of variables emerges on the impact of teleworking through direct or indirect mechanisms and a number of interactions with worker’s characteristics. First, the features of the environment matter, as it affects well-being, for example, depending on a better digital infrastructure, access to daylight and sufficient space. Second, it is not only a question of “where” we telework, but also “how much”. The advantages of a hybrid mode seem to be emerging to avoid an excessive lack of in-person social interaction, while offering greater flexibility in organizing daily life and reducing commuting times. Third, beyond the modalities of teleworking *per se*, it is key to take into account how these interact with workers’ personalities, their choices and preferences, which are often dictated by the stage of life they are in, e.g., parenting and career stages. In sum, the literature suggests that a straight answer on the positive or negative effects of teleworking is neither useful nor necessary. Multiple answers are possible to unveil the specific working arrangements that makes workers’ lives better according to their different needs. It seems essential to continue research on teleworking away from the exceptional stressors of the COVID-19 pandemic, which have greatly skewed the evidence on the detrimental effects of teleworking. Planning more complex research designs using longitudinal data and network analyses could improve understanding of how teleworking is changing careers, lifestyles and social relationships.

## Introduction

What’s going on with teleworking? Governments and firms around the world have made huge use of remote working as response to the “stay-at-home” policies designed throughout the COVID-19 pandemic. This constituted a massive, global experiment in the world of work and a turning point for the lives of millions of people [1]. In many of the world’s largest economies, more than half of all workers worked from home throughout the pandemic, and many analysts today agree that teleworking is “here to stay” [2], that it represents the “new normal” [3] and, moreover, that it is set to grow in the future [4]. Far from the exceptional circumstances of the pandemic, the data has made clear its exceptional growth. In the United States, where the advent of remote working arrangements seems to be penetrating with greater force, the total number of days worked at home now steadily accounts for 27 percent of the total. This is a full four times the pre-pandemic levels, meaning that teleworking has experienced fifty years of growth in just four years [see 5 for a detailed overview of this trend]. There is thus considerable interest in research on teleworking, not only because at issue is the productivity of the most qualified segments of the economy, but especially because of its consequences for the lives and well-being of workers [6].

Regarding teleworking and well-being, the pandemic has left us with a fragmented picture of evidence, which we have systematized here following the PRISMA guidelines for scoping reviews [7]. The main objective of this paper is therefore to provide an overview of existing studies up to December 2022 in an effort (i) to map out key determinants to be taken into account in this literature. What emerged from this systematization is a complex picture of factors, as we explain, in which emerge, above all, the role played by the characteristics of the work environment [8] and the hours devoted to teleworking, hinting at the benefits of hybrid modalities rather than full-time remote vs. in-presence work [9]. This provides a picture of evidence in which one cannot fail to take into account the fact that the effects of teleworking also depend on personal choices, personalities and inclinations, and on the times of life in which teleworkers find themselves, such as parenting [10]. Moreover, as a general consideration, a rather negative legacy of the pandemic emerges from this scoping review, with an image of teleworking that is skewed toward the idea of isolation and loneliness, which is actually more a result of the “stay-at-home” policies than to teleworking *per se* [11].

Another objective of this paper that enriches this overview is (ii) to highlight the findings on those social mechanisms that have been found as mediating factors between teleworking and well-being, such as the effect on personal networks, i.e., social support, or the work-life balance. Again, as we explain, a varied array of mechanisms has emerged in which our relations with relatives and colleagues and the form in which we organize our daily lives come into play. We summarize this framework of evidence differentiating between factors that directly or indirectly affect well-being, or that simply interact in the relationship between teleworking and well-being. Hopefully, as we discuss in the final section, in this way we will be able to shed light on the strengths and weaknesses of this literature and to launch avenues of research to improve understanding of this great transformation in the world of work [12–17].

## 2. Methods

### 2.1 Inclusion criteria

The review focused on studies that look at teleworking as a determinant (predictor) of well-being markers (outcomes) in adults aged 18 and above. Following [4, p. 5], we define teleworking as “any form of organizing and/or performing work using information technology in the context of an employment contract/relationship, in which work, which could also be performed at the employer’s premises, is carried out away from those premises (…)”. Because various approaches to categorizing teleworking exist, and because teleworking activities can be grouped into different categories, we took account of studies that identify remote working regardless of the types of activities, modes, or rates of work considered, or the contexts in which they took place [18].

Well-being is also a multifaceted concept that has been addressed through a plurality of theoretical frameworks [see 19 for an overview]. In this scoping review, we discuss this concept by considering studies investigating well-being through indicators for mental health and quality of life. The rationale for the selection of these indicators lies in the fact that there is a long tradition of studies testing how mental health issues are related to exposure to screens and the use of information and communication technologies [e.g., 14,20]. On the one hand, one type of mental health indicator refers to diagnostic tools to assess Common Mental Disorders (hereafter CMD), a range of mental conditions described in the DSM-V, including disorders such as depression, anxiety disorders or insomnia, among others [21]. However, much of the literature on teleworking assesses workers’ mental health without relying on diagnostic criteria and, more importantly, without referring to severe disorders such as those considered by the DSM-V. Therefore, we have also included studies that refer to moderate mental health issues, e.g., forms of exhaustion or psychological distress, which are often measured in the literature using single item or self-reported measures of mental health as outcomes. Furthermore, given that there is a wide interest among scholars of teleworking in how it changes family dynamics and the work-life balance, we also included those research studies that used indicators relating more generally to people’s quality of life, measured through indicators of life and job satisfaction, energy and vigor, or work engagement, among others [22]. We excluded indicators relating to musculoskeletal health [23] because they mostly address medical issues unrelated to social mechanisms. Among our exclusion criteria, we opted not to include studies that reverse the relationship between teleworking (predictor) and well-being (outcome).

We included peer-reviewed studies written in English and published from 1 January 2000, to 31 December 2022. We did not consider articles published before this period because, as explained in [5], none of the key technologies that characterize teleworking today–for example, platforms such as Zoom, Webex, Drive or Dropbox–existed before 2000. However, to encompass the largest body of evidence along this period, no restrictions were set concerning the regions where the studies were carried out or the type of research design. Thus, we included in our review qualitative and quantitative studies, as well as literature reviews, e.g., scoping and systematic reviews. For a comprehensive understanding of our selection process, the detailed inclusion and exclusion criteria are provided in the Appendix A (Table A) in S1 File.

### 2.2 Information sources, search strategy and screening

We employed a combination of 57 keywords related to teleworking and well-being to search studies on this topic in three bibliographical databases: Social Sciences Citation Index (Web of Science), Sociological Abstracts (PROQUEST) and SocINDEX with full text (EBSCOhost). The selection of these databases reflected our aim of identifying the social mechanisms (e.g., social networks) that shed further light on the relationship between teleworking and well-being. As part of our search strategy, we checked the references of articles that met our inclusion criteria in case there were any additional sources we could include. A detailed description of our search strategy is available in the Appendix A in S1 File (*General search strategy*).

### 2.3 Source selection, data management and synthesis

Identified references were imported into Covidence systematic review software [24]. The three authors separately screened titles and abstracts against the inclusion criteria and established an inter-rater reliability of around 80% after screening 20% of the abstracts. Disagreements were discussed and resolved among the authors before continuing to screen full-text articles against the inclusion criteria. After the title and abstract screening, 299 papers that matched the inclusion criteria were retained for the full-text screening procedure. The three authors thus screened first 20% of these papers to ensure reliability and consistency, resolving possible disagreements, before fully screening the entire pool of selected studies.

For the 132 articles that met the inclusion criteria, relevant data from each study were extracted into an Excel spreadsheet. The data covered 29 different types of information related to the following aspects: aim of the study, type of population, sample size, research design, methods, year, geographical context, teleworking activities analyzed (e.g. intensity, environmental characteristics), methodologies used to measure such activities, well-being indicators used and their measurement, and results (which included summary statistics, key findings and recommendations). Critical appraisal tools to evaluate the studies were used, such as the Critical Appraisal Skills Programme or CASP for quantitative and qualitative studies [25], AMSTAR for systematic literature reviews [26], and the McGill Mixed Methods Appraisal Tool (MMAT) for mixed-methods designs [27].

A content analysis was performed in two rounds to synthesize the information retrieved into the Excel spreadsheet [28]. In the first round, we linked the extracted data with our broader conceptual framework, thus identifying information related to the following three categories: (1) “telework modalities”: articles providing information about the different features and modalities of teleworking activities; (2) "social mechanisms”: articles providing information about the associations and causal mechanisms linking such teleworking modalities with well-being; and (3) “well-being”: articles providing information about the markers used to measure well-being. In the second round, in which data relating to these three categories were categorized, we used an informed coding scheme to register the information separately. We marked off segments of data into the Excel spreadsheet using sub-codes–for instance, “blurred work-life”, “parenting”, or “social support”–to determine if and how teleworking was related to well-being through direct or indirect mechanisms or interaction effects with worker’s characteristics. This allowed us to move inductively towards a map of mechanisms that have sub-codes in common, thus making them distinctive from each other.

## 3. Results

### 3.1 Description of included studies

**Fig 1:**
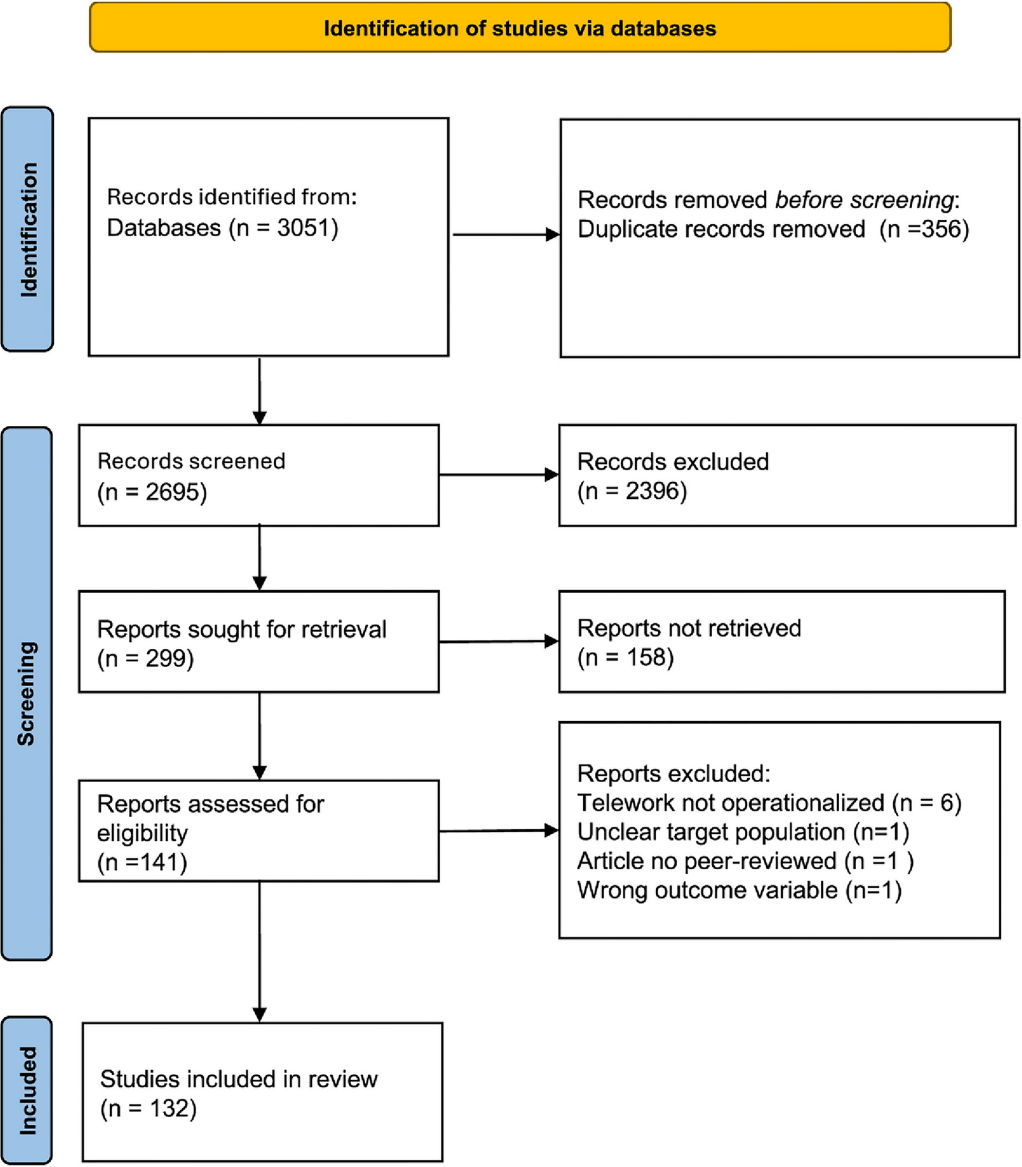
PRISMA flow diagram.

A total of 132 studies were included in this review. The selection procedure is outlined in the PRISMA flow diagram [7], depicted in Fig 1.

### 3.2. Overview of the results

Table 1 gives a descriptive overview of the included articles. Moreover, a detailed summary of these 132 studies is displayed in S2 File, including authorship, year of publication, country of study, modalities and features of teleworking investigated, measures of well-being used, methodology, sample size and main findings.

**Table 1:** Overview of the the studies.

Characteristics	Number of articles	Percentage (%)
*Publication year*		
2020–2022	105	79.5
		
2000–2019	27	20.5
*Methods*		
Literature review	12	9.1
Mixed-methods	6	4.5
Quantitative	94	71.2
Qualitative	20	15.2
		
*Sample size*		
10–100	23	19.2
101–500	35	29.2
501–1000	16	13.3
1001–5000	50	41.2
>5000	16	13.3
*Well-being outcomes*		
Mental health (including CMD)	69	57.7
Quality of life	51	42.5
*Quality assessment*		
Good	47	35.6
Moderate	52	43.3
Poor	33	25

[S2 File]

**Fig 2:**
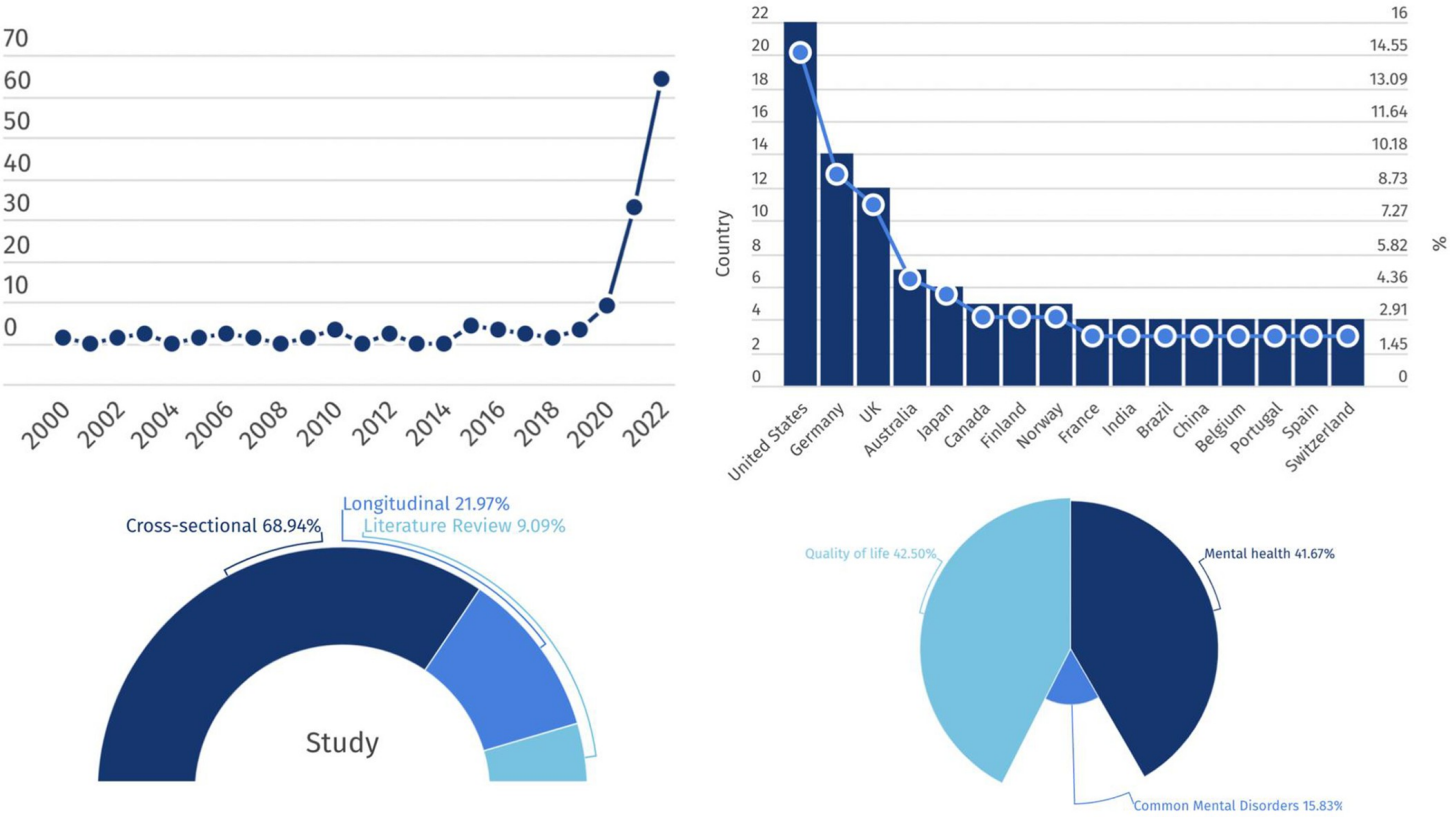
Infographic. On the top left, the number of studies by year. On the top right, the number of studies by the most represented country. On the bottom left, the percentage related to research design and literature reviews. On the bottom right, the percentage related to the well-being markers used by the screened studies.

One initial observation is the growth of studies of teleworking and well-being during the COVID-19 pandemic [Fig 2]. In total, 105 articles were published between 1 January 2020 and 31 December 2022, which represent 79.5% of our sample. Amongst the papers published in 2020, 83 explicitly concern the COVID-19 pandemic. The other 22 studies published during this time period either collected data before the pandemic, or simply lack clear information about the data that is used and therefore does not allow us to state if they are related to the pandemic.

Studies are conducted in a great number in the United States of America (22 articles), followed by Germany (14), the United Kingdom (12), Australia (7 articles), Japan (6 articles), Canada, Finland and Norway (5 articles), France, Brazil, China, India, Belgium, Portugal, Spain, and Switzerland (4 articles), Austria, The Netherlands, Poland and Sweden (3 articles), Argentina, Hong Kong, Italy, South Africa, Wales and Turkey (2 articles), Bangladesh, Chile, Denmark, Israel, Lebanon, Romania, Saudi Aurabia, Slovenia, Thailand, Vietnam, Croatia, Colombia, Ireland, Mexico, Nigeria, Taiwan, Singapore and New Zealand (1 article). In total, the scoping review analyzed articles from 44 countries. A large share of studies (99) focus on data retrieved from a single country. Only 14 studies use cross-country samples — seven studies do not specify the country were the study was held.

Of all the studies included, 94 articles have a quantitative design, 20 adopt a qualitative approach and six utilize a mixed methods design. In total, we included in the screening twelve literature reviews. Among the empirical papers, a large proportion of studies (91 articles) are cross-sectional, whereas only 29 studies use a longitudinal approach. The sample size of these studies varies greatly, partly as a result of the different methodologies used. Among the 20 qualitative studies, 16 have a sample size of less than 100 individuals [e.g. 10,29,30], 3 studies have a sample size between 101 and 500 and one article uses a sample size larger than 500 [8,31,32]. Among the 94 quantiative studies, 16 have more than 5000 individuals [e.g. 33,34], 30 have between 1000 and 5000 individuals [e.g. 35,36], 14 studies use a sample size of 501–1000 [e.g. 28,37,38], 29 papers investigated between 101 and 500 persons [e.g. 39,40], and 5 have a small sample size of less than 100 [41–45].

The quality of papers was appraised using the standardized tools previsouly mentioned, i.e., CASP, AMSTAR, and MMAT. These tools evaluate the clarity of objectives and research questions and the correct operationalization of the variables or procedure. Additionally, they assess the sampling method and sample size. Of the total, 33 studies were rated as having weak quality, 52 as moderate, and 47 as strong. Most of the studies rank as weak due to poor sample quality and data-collection procedures. Indeed, the use of social media to recruit participants, non-representative samples, or small sample sizes reduced the quality of the research design [25–27]. Moreover, a large proportion of studies do not conform to a standardized scale but are based on their own constructs, which prevents them from drawing clear and well-established conclusions or producing comparable or reproducible results [25–27].

Teleworking modalities have been addressed using a large palette of constructs to explore their links to well-being. We identified and grouped these modalities in categories of issues addressed by scholars primarily related to the “where,” “how much” and “when” of the teleworking activity. First, on the “where”, we retain the features of the *environment* in which teleworking is carried out (i.e., equipment, infrastructure, location) [8]. Second, a large number of studies focus on the “how much”, that is, the *intensity* of teleworking activity, i.e., how many days a week or working hours [46]. Third, studies on well-being focused on the experience of teleworking before or during the COVID-19 pandemic and, particularly, the *lockdowns* [47]. More precisely, the category *environment* refers to the context in which the teleworking is performed, including all the technological devices used and the immediate environment where the teleworking is done. The *intensity* of teleworking puts the emphasis on the amount of time per week or number of days spent teleworking. This category includes studies questioning whether teleworking is performed at all, comparing in-presence, hybrid or fully-remote workers. With teleworking during *lockdowns* we also refer to a large cluster of studies that explore telework issues during the COVID-19 pandemic and the “stay-at-home” policies.

Overall, this scoping review thus considers quantitative and qualitative studies which investigates well-being through a multiplicity of research angles, thus operationalizing it differently through diagnostic criteria, simple single items or more in-depth qualitative inquiries. Among studies related to mental health issues (69 articles), 19 prices of research use diagnostic criteria to investigate Common Mental Disorders (CMD), specifically the emergence of anxiety, insomnia and depression disorders. An example of an instrument used in these studies is the Generalized Anxiety Disorder Scale (GAD) [34,48]. In addition to the investigation of these CMD, a large share of studies focus on mental health issues that do not meet the DSM-V criteria, such as forms of moderate psychological distress, fatigue, exhaustion or burnout, among others (50 articles). Some examples of instruments used in this group of studies are, for example, the Perceived Stress Scale (PSS) [39] or the Burnout Bullying Inventory (BBI) [49]. We labelled *quality of life* a large cluster of studies focusing on indicators of life and work satisfaction, work engagement, or work-life balance (51 items). Instruments used in quantitative research among these studies are, for example, the Utrecht Work Engagement Scale (UWES) [e.g. 13,50] or the Michigan Organizational Assessment Questionnaire–Job Satisfaction Subscale (MOAQ-JSS) [e.g. 51,52]. The complete list of instruments and scales used from the studies can be found in S2 File.

### 3.3. The complex relationship between teleworking and well-being

The link between teleworking and well-being spans many essential areas of individual life. It is a link that directly concerns the organization of one’s daily life, the quantity and quality of one’s interactions with one’s colleagues, as well as time spent at home with one’s family. It also involves mobility and daily commuting, or the ability to make time for self-care and leisure. Our content analysis reconstructs this framework of factors by first isolating the different modalities and temporalities of teleworking: the environment in which it is carried out (“where”), its intensity (“how much”) and whether it occurred before or during the pandemic (“when”). Mechanisms emerge that directly link teleworking and well-being, but also mediating factors (e.g., → personal networks →) or interactions with the characteristics of teleworkers, such as gender and their personality. In the following subsections, we summarize the main arguments that have emerged from the literature; In Table 2 we propose six “takeaway” messages from each section.

**Table 2:** Six “takeaway” messages.

	Section	“Takeaway” messages
General remarks	**3.3.1**	We found a very divided literature between positive and negative effects
Covid-19	**3.3.2**	Many people were unprepared for the abrupt transition to teleworking
Work Environment	**3.3.3**	The “where” matters: a better telework environment increases well-being
Intensity	**3.3.4**	Hybrid is better: less commuting, more time to oneself and you avoid isolation
Personal networks	**3.3.5**	Less social support, conflicts and interruptions and more diversity of contacts
Workers’ features	**3.3.6**	It’s not just “where” and “how much” we telework, but also “who” the teleworker is

#### 3.3.1 A very divided literature on positive and negative effects

A very divided literature on the positive and negative aspects of teleworking emerges from this systematization. In total, we counted 89 studies that found positive effects [53–55], while 112 found negative effects [e.g. 28,56,57], 9 studies found a reversed U-shape association [58,59], and 15 found mixed associations [e.g. 60,61], both positive and negative, depending on the type of indicator. The frequency of these associations gives us a simple overview of this division. The influence that “stay-at-home” policies have had in skewing this literature towards the negative effects of isolation is very clear [62–68]. Even before the pandemic, working remotely was associated with feelings of isolation [53], but one possible strategy for coping with the lack of interaction was to maintain relationships in other areas of life [69]. Due to the lockdowns, the ability to compensate for this loss of physical contact with other areas of socialization was impossible [10,70]. In general, positive effects are proportionally more present in the literature before COVID-19 [32,45,71–77].

#### 3.3.2. Uncertainty at the the outbreak of the COVID-19 pandemic

One negative effect that clearly emerges is related to the uncertainty that resulted from the outbreak of the COVID-19 pandemic and the abrupt switch to teleworking [11,12,34,78–80]. Much of the population was unprepared for the challenges and obligations inherent in the teleworking experience [57,79,81]. For example, [79] found that individuals who transitioned from never working from home to full-time remote working reported lower mental well-being. The deterioration of mental health has been associated with the need to integrate new tasks, logistical problems, unclear expectations about the work, the reorganization of team working [78] and the need develop new IT skills [82], especially for older workers [68].

#### 3.3.3. The work environment matters, and it affects well-being

Scholars analysed the importance of the teleworking environment in 16 studies [e.g. 36,83,84]. The results highlight the negative effects of an inadequate infrastructure, characterized by problems such as poor Internet connectivity [10,29,85], frequent interruptions [31,86,87] and physical discomfort [48]. Specific elements, such as the absence of outdoor spaces, natural light, greenery and outdoor views, have been identified as important in promoting positive well-being [36,88,89]. In addition, noise levels have been highlighted as factors that negatively affect the quality of life [36,86]. Research suggests that an adequate infrastructure improves concentration and job satisfaction [9,36,88]. Sub-optimal infrastructure for teleworking has also taken the form of the sudden and unexpected switch to teleworking during the pandemic [86,90]. In contrast, when the switch to teleworking is voluntary or occurs outside of emergency situations, individuals invest time and resources in ensuring a suitable working environment, thereby improving well-being [77]. The features of the environment can have a direct positive association with well-being and can also generate a number of indirect mechanisms because sufficient infrastructure: for instance, having a separate work room may prevent the work-life boundary from becoming blurred and avoid interruptions [10,89].

#### 3.3.4. Hybrid is better? On telework intensity and the advantages of more autonomy and flexibility

In 9 studies, the intensity of teleworking has been found to have a quadratic (inverted U-shape) effect on the well-being of the teleworkers [9,40,91–94]. This means that, rather than working all the time remotely or being all the time in-presence, hybrid formulas have proved to be more advantageous in these studies. For example, [92,93] found that a curvilinear relationship exists between the extent of teleworking and life satisfaction. Both too little and too much remote work has been associated with lower job satisfaction, while moderate intensity found to be optimal [93]. In two other studies, [40,94] also found an inverted U-shaped relation with regard to self-reported mental health and the incidence of sleep disturbance. The advantages of hybrid modalities over full-time remote working include avoiding the lack of physical contact and reducing the risk of blurring work-life boundaries [39,48,95]. Compared to fully present workers, teleworking in hybrid form decreases commuting time, which in ten studies is clearly associated with reduced stress [17,19,54,60,76,96,97]. In addition, [8,11,12,98] show the benefits of having more autonomy and flexibility to improve the work-life balance by allowing workers to organize their days better, having more family time and engagement in self-care and healthy lifestyle behavior.

#### 3.3.5. The mediating role of personal networks: loneliness, social support, interruptions and conflicts

A total of 63 studies point to the role played by personal networks in mediating the relationship between teleworking and well-being. This means that teleworking impacts the way people spend time with their social relationships, particularly family members and colleagues, which in turn affects their mental health and quality of life. The literature is also quite divided on these relational issues, highlighting both positive and negative effects. Overall, this body of literature addresses issues such as loneliness, social support, interruptions and conflicts.

- *Loneliness* is among the most frequently highlighted issues (29 articles). The sense of isolation that emerges due to reduced face-to-face interactions is negatively correlated with life satisfaction [31,99,100]. However, again it is worth emphasising that much of this evidence was produced in the difficult context of lockdowns [60,82], and it seems difficult to draw lessons about teleworking under normal circumstances and for all those who do not work remotely full time [12].
- Twenty-nine studies examine *social support*, such as emotional aid, instrumental help and sense of belonging. They mainly show that teleworking decreases the perception of social support, which in turn negatively affects well-being [91,92,96]. However, 2 studies [51,101] show that teleworking fosters the development of a sense of community by enabling communication with members of the organization who are physically distant (e.g., due to the geographical distribution of company employees) [11,53]. Four studies [11,19,53,102] show that having more digital contacts enables teleworkers to diversify their networks, although it decreases communication with superiors [33]
- Eight articles focus on *work interruptions*. [53,87,103] show that teleworking can reduces interruptions from colleagues, but also [102] that workimg at home can increase interruptions from family members. The latter mechanism is also related to the COVID-19 pandemic and, as already mentioned, to an abrupt transition to teleworking and the inadequacy of the working environment (e.g. too small and inadequate spaces) [36,86].
- *Conflicts and forms of control*. Another issue concerns negative relations at work [13,81,104]. According to [105] teleworking can allows one to distance oneself from difficult ties, thus reducing negative experiences such as mobbing and bullying [13]. Teleworking fosters digital interaction, which seems to facilitate control and supervision in [81,88,104]. The effects of interacting with bosses and managers has been found to have an inverted U-shape in [81,91,104,106], suggesting that not only is a lack of interaction harmful, but also that an excessive amount can be detrimental, since it is experienced as a form of control.

#### 3.3.6. Who benefits from teleworking? a question of personality and life stages

Personal characteristics may amplify or mitigate the effects of teleworking. Eleven studies focus on the role played by worker’s personality [36,69,107]. Studies of the so-called “big five” shows that neuroticism among teleworkers has been treated as a predictor of work exhaustion, while agreeableness and conscientiousness have been identified as protective factors for well-being [102,108]. Individuals with high levels of emotional stability appear to be better able to satisfy the autonomy gained by teleworking, which translates into positive outcomes. The literature therefore emphasizes workers’ inclinations and preferences to explain that some individuals need to feel more structure and direction [82], while others feel overburdened by the social control and demands that technological tools entail [39,109]. In sum, workers’ personalities matter.

Gender differences and parenting are also important. The difficulties experienced during the pandemic by parents being forced into the simultaneous schooling of children clearly emerge in [10,110]. Most of the 14 studies dealing with gender issues thus reflect this difficult period, showing how working from home blurred role boundaries to a greater extent for women [19], exacerbating the genderized organization of domestic and childcare work, and increasing the mental overload for women [48,57,68,85]. Work schedule and location flexibility are shown to help working mothers in [105], but overall evidence on gender should be reinforced under normal conditions.

Age and professional status also plays a role in linking teleworking to well-being. Teleworking has been shown to worsen older adults’ life satisfaction [58]. Overall, an inverted J-shaped effect was found: that is, belonging to a younger generation is a protective factor in the context of teleworking, whereas being aged over fifty was found to interact negatively among teleworkers [58]. The underlying mechanism is a lower level of adaptability to the strong penetration of technology in the daily lives of older adults [79,88,97]. The professional position of the worker also plays a significant role. Three studies suggest that low-skilled workers are less satisfied in the teleworking context than bosses and managers [70,83,87].

## 4. Discussion and conclusions

This scoping review shows the great interest in the study of teleworking today. As many as 79.5 percent of the studies in our sample addressed the links between teleworking and well-being as of January 2020, especially as a result of the huge reliance on remote working during “stay-at-home” policies. It is therefore important to keep in mind that much of the literature we systematized in this scoping review was produced in this emergency context. The result is a fragmented picture of the evidence, of which we offer a general overview to draw lessons for the future. This we believe will help in light of the great divide we found between studies that emphasize respectively the positive and negative effects of teleworking. This is a division that invites reflection on causes and specifics, in the awareness that a *tout court* response seems neither useful nor necessary. There are some pitfalls in teleworking, but equally clear are the opportunities that can be exploited to improve workers’ lives [8].

From the difficult context in which much of the evidence has been produced during the pandemic, an image of teleworking has emerged that is often associated with isolation and loneliness [100]. This association should be kept in mind because studies even before the pandemic had laid bare the dangers of a lack of physical contact and social interaction [53]. In this sense, the literature seems to highlight the benefits of hybrid modalities, showing a curvilinear relationship with well-being, which seems to suggest a more balanced path between in-person work and fully remote working [91]. A meta-analysis on a smaller and more homogeneous number of studies may provide more precise answers to the hybrid arrangements that should be prioritized [74].

It is clear from this scoping review that a favorable infrastructure, good Internet connectivity, an absence of noise and access to natural light, greenery and views from outside all promote the well-being of those who telework [10]. Being unprepared for the digital logistics of teleworking was one of the factors most cited as detrimental to well-being, this also being a concern for firms wishing to prepare and train their employees for teleworking [82]. Among the mediating effects that have emerged most clearly as beneficial to well-being is a reduction in commuting times [17,19,96] and greater autonomy and flexibility for organizing one’s daily life [8,11]. Again, hybrid arrangements seem to anticipate some pitfalls, for example, that flexibility flows into forms of self-exploitation [12,98] and blurs work-life boundaries [48,95].

Relationships with family members and colleagues appeared to be among the key mechanisms in this literature. Associated with isolation and loneliness, teleworking is consequently linked to lower perceptions of social support, with a negative impact on well-being [91,96]. Evidence remains to be strengthened away from pandemic stressors and to be evaluated not only for fully remote workers, but also for hybrid workers. Among the relational aspects to be evaluated, there is also positive evidence of how, precisely because of digital interactions, teleworking provides an opportunity to diversify one’s contacts, often connecting with geographically distant colleagues and fostering a sense of community [51,101]. In addition, although less studied, it is interesting to note that teleworking has also been found to mitigate conflict, forms of bullying and mobbing [13], thus allowing distancing from difficult relationships and reducing interruptions and distractions at work [53,87,103].

This paper contributes to the literature on teleworking by providing this overview of mechanisms, both direct and indirect, and making it clear that interacting with them are mainly workers’ personal characteristics, such as personality traits and gender differences. It was difficult for many young relatives to work from home during the pandemic and simultaneously follow online schooling [58], but today teleworking can offer them the autonomy and flexibility that seems to benefit family life [8,11]. Some interesting evidence also points to a relationship between age and well-being during teleworking, whereby those who benefit most from teleworking are those who are neither at the beginning nor at the end of their careers. Evidence must be reinforced to better, understand how teleworking and well-being depends on employment status and hierarchies [70,83,87].

This scoping review has some limitations. First, we included studies written only in English, excluding the gray literature, which could certainly have excluded a substantial body of valuable research. The exclusion of physical health issues is also a limitation of this scoping review. In addition, new evidence is now emerging on the relationship between teleworking and well-being under normal circumstances, and an updated review of this work should be done. Partly as a consequence of the concentration of studies in the pandemic years, much research was designed as rapid responses to the health emergency [60], and thus had some methodological limitations, such as sampling strategies based on online recruitment and small samples. However, there is no shortage of articles in our sample that are of good or excellent quality, putting us in a position to draw some more general lessons about the future of teleworking. Finally, the inclusion of a large number of qualitative and quantitative studies made it difficult to compare the evidence across studies and to redetermine effect sizes for each of the mechanisms we identified. It seems appropriate to plan a meta-analysis on a smaller number more homogeneous quantitative studies to go more deeply into the analysis of these mechanisms.

More longitudinal studies of teleworking seem necessary in the future to look at its effects on well-being in the broader context of professional careers. Furthermore, given the centrality of studies focusing on the effects of teleworking on social relationships, the use of social network analysis (SNA) would provide structural information on personal networks, which is totally lacking in the literature. Moreover, since the work environment seems to be so important in understanding the effects on well-being, it is also important to understand how remote working not only takes place at home, but everywhere today, and how smartphones are heling facilitate this growth. In conclusion, this scoping review on teleworking and well-being reveals a complex and multifaceted relationship. The COVID-19 pandemic has had a significant impact on interest in studies of teleworking, and most research has focused on this period. We hope that this review will serve the future of teleworking research, guiding workers, institutions and firms to govern this important transition that the world of work is experiencing.

## Supporting information

S1 Checklist Preferred Reporting Items for Systematic reviews and Meta-Analyses extension for Scoping Reviews (PRISMA-ScR) checklist. (DOCX)

S1 File (DOCX)

S2 File (XLSX)
