# Correction: What’s going on with teleworking? a scoping review of its effects on well-being

**DOI:** 10.1371/journal.pone.0344475

**Published:** 2026-03-09

**Authors:** The PLOS One Staff

The word “a” should be capitalized in the article title. The correct title is: What’s going on with teleworking? A scoping review of its effects on well-being. The correct citation is: Vacchiano M, Fernandez G, Schmutz R (2024) What’s going on with teleworking? A scoping review of its effects on well-being. PLoS ONE 19(8): e0305567. https://doi.org/10.1371/journal.pone.0305567

There are errors in the author affiliations. The correct affiliations are as follows

Mattia Vacchiano^1,2^, Guillaume Fernandez^1,2^, Rita Schmutz^2,3^

**1** Department of Sociology, University of Geneva, Geneva, Switzerland, **2** Swiss Centre of Expertise in Life Course Research LIVES, Geneva, Switzerland, **3** Institute of Social Sciences, University of Lausanne, Lausanne, Switzerland.

The publisher apologizes for the error.
