# Supplementary material for: What’s going on with teleworking? a scoping review of its effects on well-being
Source: PLoS One. 2024 Aug 19;19(8):e0305567. doi: 10.1371/journal.pone.0305567 (PMC11332997; doi:10.1371/journal.pone.0305567)
Supplement: S1 File — (DOCX) [file pone.0305567.s002.docx]

**Appendix A**

**Table A: Inclusion and exclusion criteria**

| **Include** | **Exclude** |
| --- | --- |
| - Studies that look at telework activities as a predictor. - Telework activities is defined as any work carried out outside the employer’s premises regardless of the technology used. - Studies that include telework activities at any rate, including full time or part-time activities. - Telework activities can take place in any setting, by any professional categories, in any segment of the labour market. | - Studies that look at telework activity as an outcome variable. |
| - Studies that consider wellbeing as an outcome. - Include studies that say the outcome variable is mental health or a type of common mental disorder, regardless of how it is measured. | - Studies that look at wellbeing as a predictor or mediator variable. |
| - Peer reviewed-scientific studies using any empirical research method or design (including cross-sectional/longitudinal approaches). - Include possible other scoping/ systematic reviews if they exist | - Grey literature, or literature reviews (that do not use an empirical method) |
| - Studies including the working population (age 18 and over), in any setting. | - Studies focused on any other population. |
| - Studies published between 2000 and 2022. | - Studies published before 2000 or after 2022. |
| - Studies written in English | - Studies written in any other language. |

**Appendix B**

*General search strategy*

We searched the following electronic databases for relevant studies: Social Sciences Citation Index, Sociological Abstracts, SocINDEX with full text.

Keywords:

(“working from home” OR “telework” OR “telecommuting” OR “remote work” OR “distance work” OR flexiplace OR “virtual work” OR “distributed work” OR “flexible work” OR “part-time telecommuting” OR “smart work” OR “agile work” OR “mobile work” OR “ICT-based mobile work” OR “ICT-based work”)

AND

(“Health” OR “Mental Health” OR “Mental Distress” OR “Mental well-being” OR "subjective well-being" OR "Emotional well-being" OR "Ill-being" OR “Psychological stress” OR “Psychological well-being” OR "psychological distress" OR “Distress” OR “Stress” OR “Anxiety” OR “Burnout” OR “Depression” OR “Disengagement” OR “Emotional exhaustion” OR Exhaustion OR Fatigue OR “Job satisfaction” OR "Satisfaction with life" OR "work satisfaction" OR "euphoria" OR “affection” OR “joy” OR “sadness” OR “worry” OR “frustration” OR “helplessness” OR "positive mood" OR "depressive “mood" OR "emotional stability" OR “optimism” OR "positive emotion" OR "negative emotion" OR “negativity” OR “resilience” OR "self-esteem" OR "personal growth" OR “Sleep deprivation” OR “Sleeping problem*”)

Web of Science: Social Sciences Citation Index

| **Search** | **Search Terms** | **Results** |
| --- | --- | --- |
| S3 | S1 AND S2 | 2,533 |
| S2 | TS=(“working from home” OR telework OR “telecommuting” OR “remote work” OR “distance work” OR flexiplace OR “virtual work” OR “distributed work” OR “flexible work” OR “part-time telecommuting” OR “smart work” OR “agile work” OR “mobile work” OR “ICT-based mobile work” OR “ICT-based work”) | 7,115 |
| S1 | TS= (“Health” OR “Mental Health” OR “Mental Distress” OR “Mental well-being” OR "subjective well-being" OR "Emotional well-being" OR "Ill-being" OR “Psychological stress” OR “Psychological well-being” OR "psychological distress" OR Distress OR Stress OR “Anxiety” OR “Burnout” OR “Depression” OR “Disengagement” OR “Emotional “exhaustion” OR “Exhaustion” OR “Fatigue” OR “Job satisfaction” OR "Satisfaction with life" OR "work satisfaction" OR "euphoria" OR “affection” OR “joy” OR “sadness” OR “worry” OR “frustration” OR “helplessness” OR "positive mood" OR "depressive mood" OR "emotional stability" OR “optimism” OR "positive emotion" OR "negative emotion" OR “negativity” OR “resilience” OR "self-esteem" OR "personal growth" OR “Sleep deprivation” OR “Sleeping problem*”) | 5,529,385 |

PROQUEST: Sociological Abstracts

| **Search** | **Search Terms** | **Results** |
| --- | --- | --- |
| S3 | S1 AND S2 | 286 |
| S2 | NOFT (“working from home” OR “telework” OR “telecommuting” OR “remote work” OR “distance work” OR “flexiplace” OR “virtual work” OR “distributed work” OR “flexible work” OR “part-time telecommuting” OR “smart work” OR “agile work” OR “mobile work” OR “ICT-based mobile work” OR “ICT-based work”) | 1,154 |
| S1 | NOFT (“Health” OR “Mental Health” OR “Mental Distress” OR “Mental well-being” OR "subjective well-being" OR "Emotional well-being" OR "Ill-being" OR “Psychological stress” OR “Psychological well-being” OR "psychological distress" OR Distress OR Stress OR “Anxiety” OR “Burnout” OR Depression OR Disengagement OR “Emotional exhaustion” OR Exhaustion OR Fatigue OR “Job satisfaction” OR "Satisfaction with life" OR "work satisfaction" OR "euphoria" OR affection OR joy OR sadness OR worry OR frustration OR helplessness OR "positive mood" OR "depressive mood" OR "emotional stability" OR optimism OR "positive emotion" OR "negative emotion" OR negativity OR resilience OR "self-esteem" OR "personal growth" OR “Sleep deprivation” OR “Sleeping problem*”) | 326,047 |

EBSCOhost: SocINDEX with full text

| **Search** | **Search Terms** | **Results** |
| --- | --- | --- |
| S4 | S1 OR S2 OR S3 | 232 |
| S3 | TI (Health OR “Mental Health” OR “Mental Distress” OR “Mental well-being” OR "subjective well-being" OR "Emotional well-being" OR "Ill-being" OR “Psychological stress” OR “Psychological well-being” OR "psychological distress" OR Distress OR Stress OR Anxiety OR Burnout OR Depression OR Disengagement OR “Emotional exhaustion” OR Exhaustion OR Fatigue OR “Job satisfaction” OR "Satisfaction with life" OR "work satisfaction" OR "euphoria" OR affection OR joy OR sadness OR worry OR frustration OR helplessness OR "positive mood" OR "depressive mood" OR "emotional stability" OR optimism OR "positive emotion" OR "negative emotion" OR negativity OR resilience OR "self-esteem" OR "personal growth" OR “Sleep deprivation” OR “Sleeping problem*”) AND (“working from home” OR telework OR telecommuting OR “remote work” OR “distance work” OR flexiplace OR “virtual work” OR “distributed work” OR “flexible work” OR “part-time telecommuting” OR “smart work” OR “agile work” OR “mobile work” OR “ICT-based mobile work” OR “ICT-based work”) | 20 |
| S2 | AB (Health OR “Mental Health” OR “Mental Distress” OR “Mental well-being” OR "subjective well-being" OR "Emotional well-being" OR "Ill-being" OR “Psychological stress” OR “Psychological well-being” OR "psychological distress" OR Distress OR Stress OR Anxiety OR Burnout OR Depression OR Disengagement OR “Emotional exhaustion” OR Exhaustion OR Fatigue OR “Job satisfaction” OR "Satisfaction with life" OR "work satisfaction" OR "euphoria" OR affection OR joy OR sadness OR worry OR frustration OR helplessness OR "positive mood" OR "depressive mood" OR "emotional stability" OR optimism OR "positive emotion" OR "negative emotion" OR negativity OR resilience OR "self-esteem" OR "personal growth" OR “Sleep deprivation” OR “Sleeping problem*”) AND (“working from home” OR telework OR telecommuting OR “remote work” OR “distance work” OR flexiplace OR “virtual work” OR “distributed work” OR “flexible work” OR “part-time telecommuting” OR “smart work” OR “agile work” OR “mobile work” OR “ICT-based mobile work” OR “ICT-based work”) | 210 |
| S1 | SU (Health OR “Mental Health” OR “Mental Distress” OR “Mental well-being” OR "subjective well-being" OR "Emotional well-being" OR "Ill-being" OR “Psychological stress” OR “Psychological well-being” OR "psychological distress" OR Distress OR Stress OR Anxiety OR Burnout OR Depression OR Disengagement OR “Emotional exhaustion” OR Exhaustion OR Fatigue OR “Job satisfaction” OR "Satisfaction with life" OR "work satisfaction" OR "euphoria" OR affection OR joy OR sadness OR worry OR frustration OR helplessness OR "positive mood" OR "depressive mood" OR "emotional stability" OR optimism OR "positive emotion" OR "negative emotion" OR negativity OR resilience OR "self-esteem" OR "personal growth" OR “Sleep deprivation” OR “Sleeping problem*”) AND (“working from home” OR telework OR telecommuting OR “remote work” OR “distance work” OR flexiplace OR “virtual work” OR “distributed work” OR “flexible work” OR “part-time telecommuting” OR “smart work” OR “agile work” OR “mobile work” OR “ICT-based mobile work” OR “ICT-based work”) | 70 |
